# The Immuno-Modulatory Activities of Pentaherbs Formula on Ovalbumin-Induced Allergic Rhinitis Mice via the Activation of Th1 and Treg Cells and Inhibition of Th2 and Th17 Cells

**DOI:** 10.3390/molecules27010239

**Published:** 2021-12-31

**Authors:** Peiting Li, Miranda Sin-Man Tsang, Lea Ling-Yu Kan, Tianheng Hou, Sharon Sze-Man Hon, Ben Chung-Lap Chan, Ida Miu-Ting Chu, Christopher Wai-Kei Lam, Ping-Chung Leung, Chun-Kwok Wong

**Affiliations:** 1 State Key Laboratory of Research on Bioactivities and Clinical Applications of Medicinal Plants, Institute of Chinese Medicine, The Chinese University of Hong Kong, Hong Kong, China; peiting@link.cuhk.edu.hk (P.L.); sinmantsang@cuhk.edu.hk (M.S.-M.T.); 1155122453@link.cuhk.edu.hk (L.L.-Y.K.); szemanhon@cuhk.edu.hk (S.S.-M.H.); benchan99@cuhk.edu.hk (B.C.-L.C.); pingcleung@cuhk.edu.hk (P.-C.L.); 2 Department of Chemical Pathology, The Chinese University of Hong Kong, Prince of Wales Hospital, Hong Kong, China; houth@g.ucla.edu (T.H.); idachu@cuhk.edu.hk (I.M.-T.C.); 3 Department of Microbiology, Immunology, and Molecular Genetics, University of California, Los Angeles, Los Angeles, CA 90095, USA; 4 Faculty of Medicine and State Key Laboratory of Quality Research in Chinese Medicines, Macau University of Science and Technology, Macau, China; wklam@must.edu.mo; 5 Li Dak Sum Yip Yio Chin R & D Centre for Chinese Medicine, The Chinese University of Hong Kong, Hong Kong, China

**Keywords:** allergic rhinitis, Pentaherbs formula, T cells, cytokines, nasal epithelium

## Abstract

Allergic rhinitis (AR) is a highly prevalent allergic disease induced by immunoglobulin (Ig) E-mediated hypersensitivity reaction at the nasal epithelium against inhaled allergens. Previous studies have demonstrated that Pentaherbs formula (PHF), a modified herbal formula comprising five herbal medicines (*Flos Lonicerae*, *Herba Menthae*, *Cortex Phellodendri*, *Cortex Moutan* and *Rhizoma Atractylodis*), could suppress various immune effector cells to exert anti-inflammatory and anti-allergic effects in allergic asthma and atopic dermatitis. The present study aimed to further determine the anti-inflammatory activities of PHF in an ovalbumin (OVA)-induced AR BALB/c mouse model. Nasal symptoms such as sneezing and nose rubbing were recorded and the serum total IgE and OVA-specific IgG1, as well as interleukin (IL)-4, IL-5, IL-10, IL-13, chemokines CXCL9 CXCL10, and tumor necrosis factor (TNF)-α concentrations in nasal lavage fluid (NALF) were measured during different treatments. Effects of PHF on the expression of inflammatory mediators in the sinonasal mucosa were quantified using real-time QPCR. PHF was found to suppress allergic symptoms, infiltration of inflammatory cells, and hyperplasia of goblet cells in the nasal epithelium of the OVA-induced AR mice. PHF could reduce OVA-specific IgG1 level in serum, and TNF-α and IL-10 in nasal lavage fluid (NALF), significantly up-regulate the splenic regulatory T (Treg) cell level, increase the Type 1 helper T cell (Th1)/Type 2 helper T cell (Th2) ratio, and reduce the Th17 cells (all *p* < 0.05). PHF could also alleviate in situ inflammation in sinonasal mucosa of OVA-induced AR mice. In conclusion, oral treatment of PHF showed immuno-modulatory activities in the OVA-induced AR mice by regulating the splenic T cell population to suppress the nasal allergy symptoms and modulating inflammatory mediators, implicating that PHF could be a therapeutic strategy for allergic rhinitis.

## 1. Introduction

Allergic rhinitis (AR) is a common allergic disease in the nasal mucosa that is mediated by the specific immunoglobulin (Ig)-E with symptoms including rhinorrhea, nasal congestion, sneezing and nose rubbing [1]. These symptoms can reduce the quality of life of AR patients. The prevalence of AR has been increasing dramatically worldwide, affecting about 10–30% of adults and 40% of children [2]. AR patients usually sustain allergen sensitization against house dust mite, animal dander, plant pollen, and mold [3]. Type 1 helper T cells (Th1) for cell-mediated immunity and Type 2 helper T cells (Th2) for humoral immunity are two distinct types of Th cells with distinct cytokine patterns [4]. The imbalance between Th1 and Th2 cells has been recognized as a crucial pathological mechanism of AR [5,6,7], and the Th2 predominant immune response in the Type I hypersensitivity is the classical pathogenesis of allergic rhinitis. The Th2-immunologic response is central to the IgE production for the recognition and interaction with allergens to cause eosinophilic infiltration in the nasal epithelium and the release of Th2 cytokines interleukin (IL)-4, IL-5 and IL-13, chemokines such as CCL5, and other allergic inflammatory mediators such as leukotrienes and prostaglandins [8].

Currently, AR patients mainly rely on well-established treatments, including antihistamine, anti-leukotriene, and glucocorticoid, which only ameliorate the rhinitis symptoms temporarily [5]. Therefore, there has been an urgent need for developing an effective and long-term alternative strategy for AR therapy. Traditional Chinese medicines (TCM) have been widely accepted for immunomodulatory treatment of allergic disease with fewer side effects. Previous studies have also found that acupoint herbal patching can exert more effective suppression on the nasal symptoms in AR patients compared to Western medicines [9,10]. Tsang et al. found that the Pentaherbs formula (PHF), a modified herbal formula comprising five Chinese Medicines of *Flos Lonicerae*, *Herba Menthae*, *Cortex Phellodendri*, *Cortex Moutan* and *Rhizoma Atractylodis*, at a *w*/*w* ratio of 2:1:2:2:2, showed anti-inflammatory and anti-allergic effects in mice with allergic asthma and atopic dermatitis (AD) [11,12]. Another previous study showed that the water extract of PHF contained soluble carbohydrates (65.9%), proteins (9.4%), soluble fibers (6.2%), and a small amount of insoluble dietary fibers (0.2%) [11]. High-Pressure Liquid Chromatography (HPLC) profiles have identified that concentrations of Gallic acid, chlorogenic acid, and berberine (which are the bioactive components or authentication markers for *Cortex Moutan*, *Flos Lonicerae*, and *Cortex Phellodendri*) in the water extract of PHF were 0.479%, 1.201% and 0.022% [12]. There were 644 compounds of PHF that could be identified from the Traditional Chinese Medicine Systems Pharmacology (TCMSP) database [13]. PHF has been shown to be an immuno-modulator of regulatory T helper cells (Treg) for the suppression of the infiltration of eosinophils, and reduction of the allergy-related cytokine release in allergic asthma and AD mice [11,12]. A network pharmacology study also demonstrated that PHF could suppress the AD symptoms by several signaling pathways, such as TNF signaling, IL-17 signaling, and hypoxia-inducible factor (HIF) signaling [13]. Many compounds in PHF are multi-targeting against AD-relate genes and proteins. For example, the polyphenol in PHF has antioxidant, anti-inflammatory and anti-allergic activities [13]. Our clinical study indicated that PHF could significantly alleviate the frequency of sneezing in AR patients, although there was no difference in other allergic rhinitis symptoms [14]. However, the immuno-modulatory role and mechanism of PHF in allergic rhinitis is unknown. 

**Figure 1:**
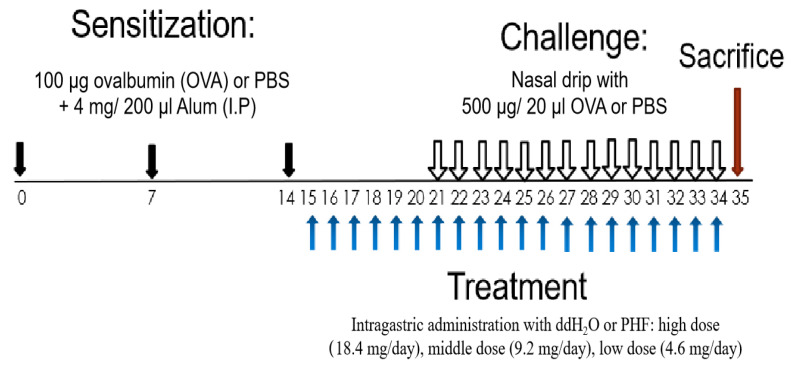
Schematic diagram of oral treatment with difference doses of PHF in the OVA-induced AR murine model. BALB/c mice (6–8 weeks old) were sensitized with 100 μg OVA together with 4 mg Alum (200 μL) by intraperitoneal injection on day 0, 7 and 14. On days 15 to 34, mice were treated with 18.4 mg, 9.2 mg and 4.6 mg PHF daily by intragastrical administration. From day 21 to 34, mice were challenged by stimulation with 500 μg/20 μL OVA that was administered intranasally 1 h after oral PHF treatment every day. Healthy controls were challenged with phosphate-buffered saline (PBS) and treated with double distilled H_2_O (ddH_2_O). All mice were sacrificed on day 35.

It is hypothesized that the compounds of PHF are multi-targeting in the AR-related gene, and PHF could have anti-allergic and anti-inflammatory properties in the AR by regulating splenic T lymphocytes and modulating the inflammatory cytokines/chemokines to alleviate the nasal symptoms of AR. An ovalbumin (OVA)-induced AR murine model was adopted, and various nasal symptoms and immunological parameters of mice upon PHF treatment were measured. The immune-regulatory mechanism of PHF in AR was further investigated to explore its therapeutic potential for treating allergic disease (Figure 1).

## 2. Results

### 2.1. Pentaherbs Formula Treatment Alleviated AR Symptoms in the OVA-Induced AR Murine Model

**Figure 2:**
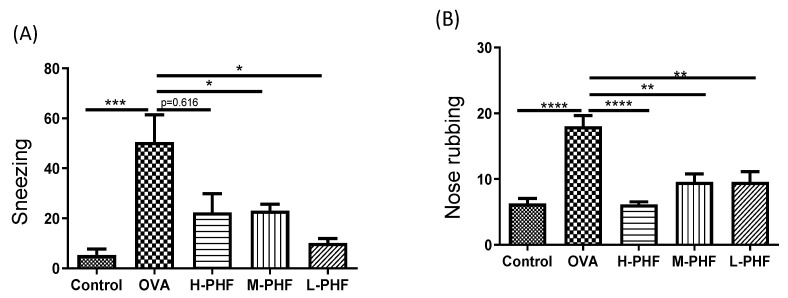
Effects of oral treatment of Pentaherbs formula (PHF) in OVA-induced AR murine model (*n* = 3–10 mice per group). The frequency of sneezing (**A**) and nose rubbing (**B**) were recorded in the first 10 min upon final OVA challenge on day 34 immediately. Results are presented as mean + SEM. * *p* < 0.05, ** *p* < 0.01, *** *p* < 0.001, **** *p* < 0.0001. Control: healthy control; OVA: OVA-induced allergic rhinitis control; H-PHF: high dose PHF (18.4 mg/day) treatment; M-PHF: middle dose PHF (9.2 mg/day) treatment; and L-PHF: low dose PHF (4.6 mg/day) treatment.

The effects of oral treatment of PHF on the frequencies of sneezing and nose rubbing during a 10-min period were recorded after the last OVA challenge on day 34. In the OVA-induced AR control group, the frequencies of sneezing (Figure 2A) and nose rubbing (Figure 2B) were significantly increased compared with the healthy control group after the final intranasal OVA challenge (*p* < 0.05). Intragastric administration of PHF significantly reduced the frequency of sneezing and nose rubbing of AR mice. These results demonstrated that oral treatment of PHF can alleviate nasal symptoms induced by OVA in the AR mice model.

### 2.2. Oral Treatment of PHF Reduced Total IgE and OVA-Specific IgG1 Levels in the Serum of the AR mice

**Figure 3:**
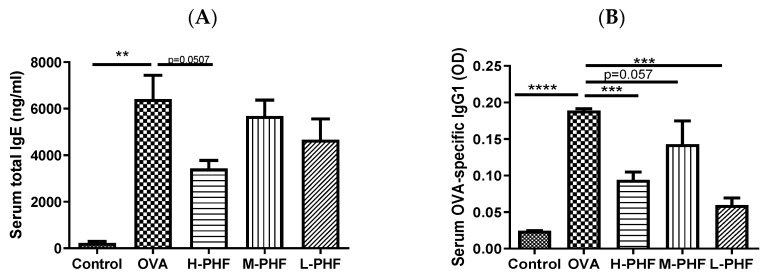
Total IgE (**A**) and OVA-specific IgG1 (**B**) levels in serum. These levels were measured by ELISA. Values represent the mean ± SEM (*n* = 3–4). ** *p* < 0.01, *** *p* < 0.001, **** *p* < 0.0001. Control: healthy control; OVA: ovalbumin (OVA)-induced allergic rhinitis control; H-PHF: high dose PHF (18.4 mg/day) treatment; M-PHF: middle dose PHF (9.2 mg/day) treatment; L-PHF: low dose PHF (4.6 mg/day) treatment.

Total IgE (Figure 3A) and OVA-specific IgG1 levels (Figure 3B) in the serum were significantly elevated in the OVA-induced AR group compared to that in the healthy control group (*p* < 0.05). When mice were intragastrically administrated different doses of PHF, the serum total IgE and OVA-specific IgG1 levels were suppressed. Especially in the high dose (18.4 mg/day) PHF treatment group, total IgE (*p* = 0.0507) and OVA-specific IgG1 levels (*p* < 0.001) were markedly reduced.

### 2.3. PHF Regulated the Proportion of Th1, Th2, Th17 and Treg Cells in the Lymphocytes of the Spleen in the OVA-Induced AR Mice

**Figure 4:**
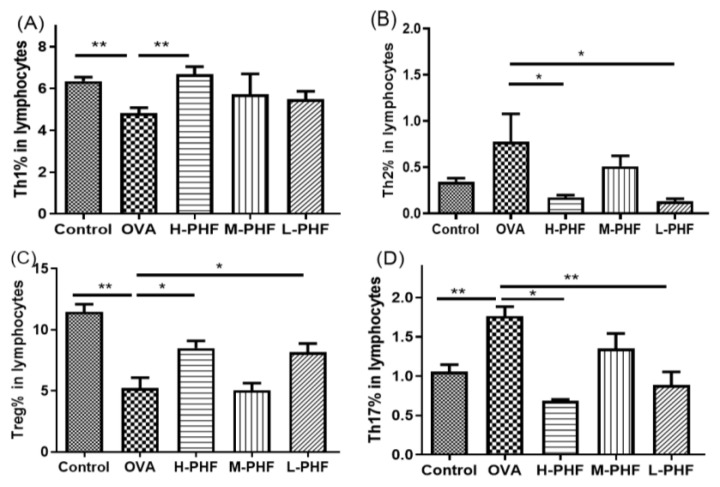
Effects of oral PHF treatment on the modulation of T cells in lymphocytes were determined by flow cytometry. The spleen was collected on day 35, 24 h after the last OVA challenge. Th1 (**A**), Th2 (**B**), Splenic CD4^+^CD25^high^ FOXP3^+^ Treg (**C**), and Th17 cells (**D**). Results are presented as mean + SEM (*n* = 3–4). * *p* < 0.05, ** *p* < 0.01. Control: healthy control; OVA: OVA-induced AR control; H-PHF: high dose PHF (18.4 mg/day) treatment; M-PHF: middle dose PHF (9.2 mg/day) treatment; L-PHF: low dose PHF (4.6 mg/day) treatment.

Mice splenic cells were cultured under specific conditions as previously described [15]. CD4^+^IFN-γ^+^ Th1 cells were found to enhance in the spleen of PHF-treated mice (Figure 4A). There was a significant reduction in CD4^+^IL4^+^ Th2 cells and CD4^+^IL-17^+^ Th17 cells in mice with oral PHF treatment compared to the OVA-induced AR control groups (all *p* < 0.05, Figure 4B,D). CD4^+^CD25^high^ FOXP3^+^ Treg in lymphocytes were significantly increased in high dose (18.4 mg/day) and low dose (4.6 mg/day) PHF treatment groups, when compared to the OVA AR control group (*p* < 0.05, Figure 4C).

### 2.4. PHF Significantly Reduced the Infiltration of Different Inflammatory Cells, Suppressed the Differentiation of Nasal Epithelial Cells, and Regulated Inflammatory Cytokines in the Nasal Lavage Fluid (NALF)

**Figure 5:**
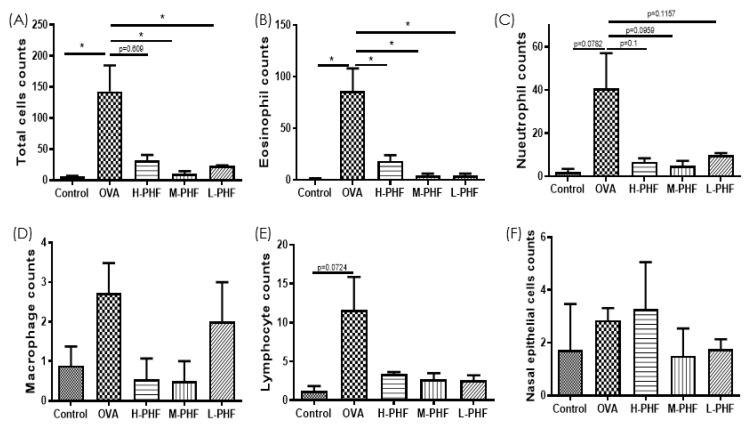
Infiltration of differential inflammatory cells and nasal epithelial cells in the NALF of the OVA-induced AR murine model with/without PHF treatment. The number of total cells (**A**), eosinophils (**B**), neutrophils (**C**), macrophages (**D**), lymphocytes (**E**), nasal epithelial cells (**F**) infiltration in the NALF were counted at the high power field (200×). Results are presented as mean + SEM (*n* = 3–4). * *p* < 0.05. Control: healthy control; OVA: ovalbumin (OVA)-induced allergic rhinitis control; H-PHF: high dose PHF (18.4 mg/day) treatment; M-PHF: middle dose PHF (9.2 mg/day) treatment; L-PHF: low dose PHF (4.6 mg/day) treatment.

**Figure 6:**
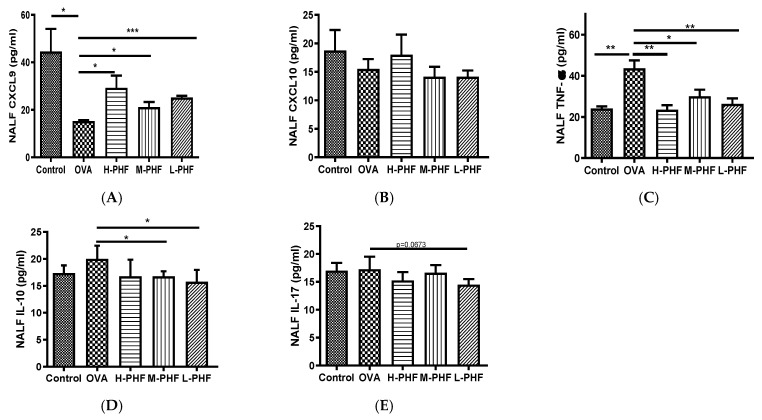
Effects of oral treatment of PHF on the chemokines and cytokines in the NALF. The levels of inflammatory chemokines/cytokines CXCL9 (**A**), CXCL10 (**B**), tumor necrosis factor (TNF)-α (**C**), interleukin (IL)-10 (**D**), IL-17 (**E**) in NALF were measured by using cytometric bead array (CBA). Results are presented as mean + SEM (*n* = 3). * *p* < 0.05, ** *p* < 0.01, *** *p* < 0.001. Control: healthy control; OVA: ovalbumin (OVA)-induced allergic rhinitis control; H-PHF: high dose PHF (18.4 mg/day) treatment; M-PHF: middle dose PHF (9.2 mg/day) treatment; L-PHF: low dose PHF (4.6 mg/day) treatment.

Total cells, nasal epithelial cells, and differential immune cells in the NALF were quantified using Kwik Diff Staining. In the OVA-induced AR group, the total cell population (Figure 5A) and eosinophils (Figure 5B) were significantly increased, while neutrophils (Figure 5C), macrophages (Figure 5D) and lymphocytes (Figure 5E) were moderately enhanced when compared to the healthy control group. After oral treatment of PHF, the number of total cells, eosinophils, neutrophils and nasal epithelial cells were reduced, but there was no significant difference in nasal epithelial cells (Figure 5F) in the NALF among the different groups. Figure 6A shows that chemokine CXCL9, which is a chemoattractant of Th1 cells [16], was significantly decreased in the OVA-induced AR group (*p* < 0.05), while different doses of PHF treatment could increase CXCL9 levels in the NALF, and high dose of PHF increased another interferon (IFN)-γ related chemokine CXCL10, which preferentially attracts activated Th1 lymphocytes [16,17]. In contrast, the levels of TNF-α (Figure 6C) were conspicuously raised in the OVA-induced AR group compared with the healthy control group, but its level reduced with the oral treatment of different doses of PHF. The level of IL-10 (Figure 6D) and IL-17 (Figure 6E) in NALF were markedly elevated in the OVA-induced AR group and suppressed by both middle and low doses of PHF.

### 2.5. PHF Significantly Reduced Infiltration of Eosinophils, PAS-Positive Goblet Cells and Mast Cells into the Nasal Epithelium

**Figure 7:**
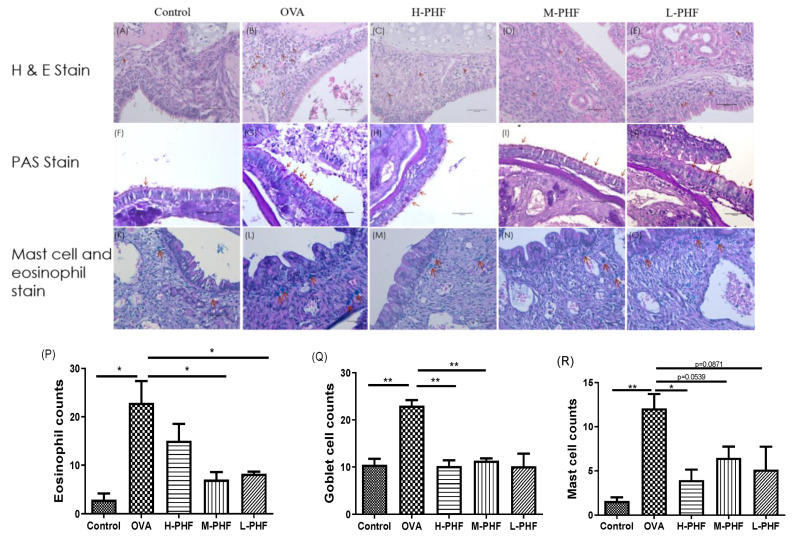
Effects of the oral treatment with PHF on the histological characteristics of the sinonasal mucosa of the OVA-induced AR murine model (*n* = 3). Representative hematoxylin and eosin staining (H&E, 200×) of the sinonasal mucosa of the AR murine model with/without PHF treatment (**A**–**E**). Red arrows denote the eosinophil infiltration in the nasal mucosa. Periodic Acid-Schiff stain (PAS, 200×) of the sinonasal mucosa of the AR murine model with/without PHF treatment. Red arrows depict the mucus-secreting goblet cells (**F**–**J**). Staining of the sinonasal mucosa of the AR murine model with/without PHF treatment was with the Eosinophil-Mast Cell staining kit (200×, **K**–**O**). Red arrows denote the distribution of mast cells. (**P**–**R**) are respectively counts of eosinophil infiltration at the sinonasal mucosa, PAS-positive goblet cells at the sinonasal mucosa, and mast cells infiltration at the sinonasal mucosa. Results are presented as mean + SEM (*n* = 3). * *p* < 0.05, ** *p* < 0.01. Control: healthy control; OVA: ovalbumin (OVA)-induced allergic rhinitis control; H-PHF: high dose PHF (18.4 mg/day) treatment; M-PHF: middle dose PHF (9.2 mg/day) treatment; L-PHF: low dose PHF (4.6 mg/day) treatment.

H&E staining (200×) of mouse sinonasal mucosa (Figure 7A–E,P) showed that the infiltration of eosinophils was significantly elevated in the OVA-induced AR group compared with the healthy control group (*p* < 0.05). After oral treatment of PHF, infiltration of eosinophils was decreased in all PHF treatment groups. Goblet cell hyperplasia is an important part of airway remodeling in allergic rhinitis, OVA treatment significantly induced the hyperplasia of goblet cells and infiltration of mast cells in the nasal mucosa of the AR group compared with the healthy control group. Importantly, PHF treatment groups also showed a reduction in the PAS-positive goblet cells (Figure 7F–J,Q) and mast cells (Figure 7K,O,R) in the nasal mucosa when compared with the OVA-induced AR group, while the OVA treatment could significantly induce the hyperplasia of goblet cells and infiltration of mast cells in the nasal mucosa compared with the healthy control group (*p* < 0.05). These results suggest that PHF can alleviate the infiltration of eosinophils and mast cells, and the hyperplasia of goblet cells in the sinonasal mucosa in the OVA-induced AR mice model.

### 2.6. PHF Alleviated In Situ Inflammation in the Sinonasal Mucosa of OVA-Induced AR Mice

**Figure 8:**
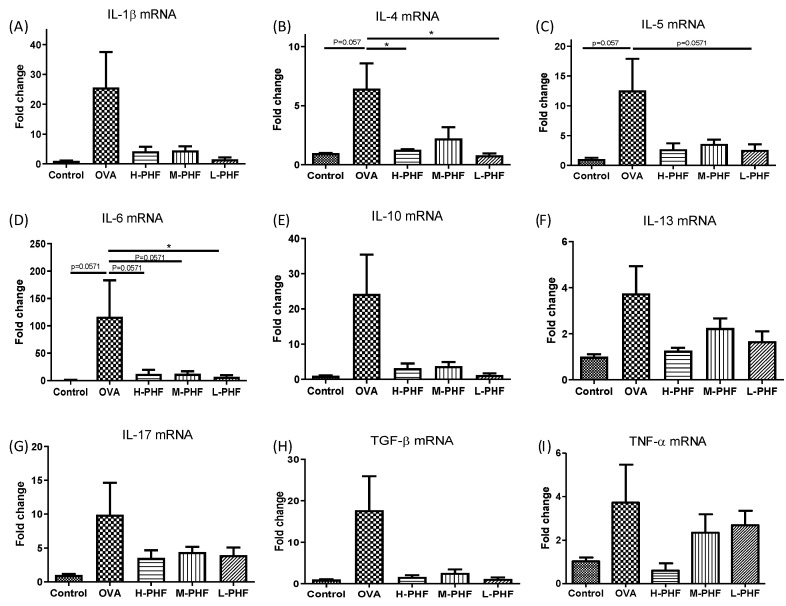
The mRNA expression levels of inflammatory cytokines in the sinonasal mucosa of OVA-induced AR mice. The mRNA expression of IL-1β (**A**), IL-4 (**B**), IL-5 (**C**), IL-6 (**D**), IL-10 (**E**), IL-13 (**F**), IL-17 (**G**), TGF-β (**H**) and TNF-α (**I**) in the sinonasal mucosa were measured by qPCR. The fold change of each gene was normalized using an internal housekeeping gene GAPDH and calculated using the cycle threshold method (2^−ΔΔ^Ct) with GAPDH. Bar charts show mean + SEM (*n* = 3, 4). * *p* < 0.05. Control: healthy control; OVA: ovalbumin (OVA)-induced AR control; H-PHF: high dose PHF (18.4 mg/day) treatment; M-PHF: middle dose PHF (9.2 mg/day) treatment; L-PHF: low dose PHF (4.6 mg/day) treatment.

As shown in Figure 8A–I, we used real-time PCR to perform in situ cytokine mRNA expression of Th2 cytokines (IL-4, IL-5, and IL-10), Th17 cytokine (IL-17), and other pro-inflammatory cytokines (IL-13, IL-1β, IL-6, TGF-β and TNF-α) of the nasal mucosa. All of these cytokines were upregulated in the sinonasal mucosa of the OVA-induced AR group when compared with the healthy control group, but the results did not fall into a trend among different doses of PHF treatment, and some of the differences did not reach statistical significance. The overall results did suggest that PHF exhibited anti-inflammatory effects by suppressing the pro-inflammatory cytokine mRNA expression, even though differential suppressive effects were not observed among different doses of PHF treatment at the protein level.

## 3. Discussion

The prevalence of AR is increasing rapidly all over the world (e.g., to 11.1–17.6% in China [18], 12–30% in the United States [19], and 23–30% in Europe [20]), with 10–30% of adults and 40% of children suffering from AR [2,21]. Pollen, animal dander, molds and house dust mite, which have large geographical variability within and between different countries, are the common allergens for AR [3]. Upon entry via the compromised nasal epithelium barrier, allergens are recognized and processed by antigen-presenting cells. The processed allergen then activates the pattern recognition receptors in the nasal epithelium, triggering a type 1 hypersensitivity reaction, including the recruitment of inflammatory cells (such as eosinophils, basophils and mast cells) and release of different allergic inflammation-related cytokines such as IL-5, IL-13 and TNF-α [22]. The AR response can be divided into two phases, the early-phase (occurring within minutes after allergen exposure) and the late-phase (developed from 2–6 h after allergen exposure) [23]. In the early phase of AR, Th2 cells play a crucial role in the production of Th2-related pro-inflammatory cytokines (IL-4, IL-5 and IL-13) that activate IgE production [23], while Th1 cells play a counteracting role by inhibiting the recruitment of eosinophils and reducing the synthesis of immunoglobulin by B lymphocytes [8,24]. A previous study has shown that AR, atopic dermatitis and allergic asthma share certain common genetic polymorphisms [25]. Therefore, these allergic diseases often co-exist in the same patient [25]. H_1_-antihistamines, corticosteroids, and leukotriene receptor antagonists are often used to alleviate allergic symptoms [26], but most of them have adverse side effects after long term use [5]. Our previous study has indicated that PHF, with multi-targeted anti-inflammatory activities, can alleviate not only the inflammatory symptoms and immune cell infiltration in local inflammatory sites in the OVA-induced allergic asthma mice, but also in the oxazolone (OXA)-mediated dermatitis-like mice [11,12,13].

PHF is an improved herbal formula with five herbal medicines. It has been reported that many compounds in PHF have multi-targeted mechanisms with anti-inflammatory and anti-allergic activities [13]. Our clinical study demonstrated that PHF could significantly suppress the frequency of sneezing in AR children with moderate-to-severe atopic dermatitis, but there was no difference in other allergic rhinitis symptoms [14]. To further explore the effect of PHF on AR, we need to establish an AR mouse model to eliminate AD interference. In this study, we investigated the immuno-modulatory activities of PHF on an OVA-induced AR mouse model. PHF was intragastrically administrated to the mice 1 week before intranasal OVA challenge. We found that PHF could prevent the development of AR by regulating the proportion of splenic T lymphocytes, inhibiting the production of immunoglobulin, modulating the inflammatory cytokines/chemokines to alleviate the nasal symptoms. The chemical composition of PHF is complex and PHF was multi-targeting on AR-related genes in the nasal mucosa. It not only downregulated the IL-17 and IL-4 gene expression but also decreased the TNF-α transcription in the nasal mucosa to suppress the level of inflammation cytokines in the sinonasal mucosa.

Distinct immunomodulatory activities were shown with different doses of PHF in the current AR murine model. High dose PHF (18.4 mg/day) showed a lower efficacy in suppressing the late phase of AR in terms of suppression of the sneezing frequency and reducing the infiltration of eosinophils in the sinonasal mucosa when compared with the middle dose (9.2 mg/day) and low dose (4.6 mg/day) PHF treatments. Nevertheless, oral treatment with high dose PHF might modulate the early phase response in AR mice, because it could significantly reduce nose-rubbing frequency, serum total IgE and OVA-specific IgG1 levels, and alleviate the imbalance of different splenic T lymphocytes. It has been found that Th1 cells play a dominant role in the early phase of the OVA-induced mice airway inflammation model [23], and oral treatment with high does PHF can significantly increase the Th1 cells but reduce the Th2 cells, and increase the Th1/Th2 ratio. High dose PHF could also suppress the splenic Th17 cells and their related cytokine IL-17 mRNA expression in the nasal mucosa [27]. Th17 has been shown to be related to Th2 production and can mediate goblet cell hyperplasia and promote mucin production in allergic disease [27]. In our study, the proportion of splenic Treg cells was significantly increased after high dose PHF treatment. Treg was found to inhibit pro-inflammatory cells and other T effector cells to suppress the allergic response in AR [28]. It has been shown that the suppressed expression of TNF-α can reduce the allergic response [29,30]. Moreover, anti-TNF-α treatment can partially restore the integrity of the nasal mucosal barrier in the house dust mite-induced AR murine model [31]. In the current study, both high dose and low dose PHF could suppress the mRNA expression of IL-4 in the nasal mucosa. Since reducing IL-4 can effectively suppress AR symptoms [32], IL-4 plays a central role of increasing barrier dysfunction in the nasal epithelium [31] and enhancing the in situ infiltration of immune cells like eosinophils, neutrophils and lymphocytes in the nasal mucosa [30]. Therefore, reduction of TNF-α and IL-4 levels by PHF might help the nasal epithelium to resist the invasion of environmental allergens and pathogens. On the other hand, high dose PHF can downregulate the mRNA expression of other allergic inflammation-related cytokines (IL-1β, IL-5, IL-6, IL-10, IL-13, TGF-β) in the nasal mucosa and increase the Th1-related chemokine CXCL9 in NALF, which plays a negative regulatory role on eosinophils in the inflammatory sites of the allergic airway and promotes CXCR3^+^CD4^+^ lymphocytes toward the Th1-type profile to diminish the allergic response [16]. In summary, the oral treatments of both middle dose PHF and low dose PHF can suppress the frequency of nose rubbing, serum total IgE and OVA-specific IgG1 levels, regulate the balance of different splenic T lymphocytes, and alleviate the inflammatory cytokines/chemokines in the sinonasal mucosa. The middle dose PHF showed a lower efficacy in decreasing the total IgE and OVA-specific IgG1 concentrations in serum but had higher efficacy in the suppression of eosinophil infiltration in the nasal mucosa. Thereby, the middle dose of PHF might modulate the late phase instead of the early phase of AR. Middle dose PHF also had higher potency in reducing the total cells, different inflammatory cells, and nasal epithelial cells in the NALF, which demonstrated that middle dose PHF can restore the barrier integrity of the nasal epithelium to suppress the infiltration of inflammatory cells. PHF also exerted anti-inflammatory activities by suppressing the mRNA expression of IL-4 in the nasal mucosa, which can alleviate in situ infiltration of immune cells like eosinophils, neutrophils, and lymphocytes in the nasal mucosa [33]. 

## 4. Materials and Methods

### 4.1. Water Extraction of Pentaherbs Formula (PHF)

The five-herbs formula comprised 10 g *Flos Lonicerae* (Jinyinhua), 5 g *Herba Menthae* (Bohe), 10 g *Cortex Moutan* (Danpi), 10 g *Rhizoma Atractylodis* (Cangshu), and 10 g *Cortex Phellodendri* (Huangbai). The raw medicines were purchased from Zi Sun (Hong Kong) Limited, Kwai Chung, Hong Kong; they were authenticated, and extracted by refluxing in boiling water as previously described [11,12]. Briefly, the Pentaherbs formula was prepared by mixing the five herbs together and refluxing the mixture in 100 °C boiling water for an hour three times. The mixture of the water crude extracts was filtered and freeze-dried into powder. 

### 4.2. Preparation of the OVA-Induced AR Mouse Model

Pathogen-free BALB/c mice (aged 6–8 weeks, 15–20 g body weight) were obtained from The Laboratory Animal Services Centre, The Chinese University of Hong Kong (Hong Kong, China). All animal studies were conducted in accordance with the guidelines of the Animal Experimentation Ethics Committee Guide for the Care and Use of Laboratory Animals, which was approved by the Animal Experimentation Ethics Committee of the Chinese University of Hong Kong.

Inbred adult BALB/c mice were sensitized by intraperitoneal (i.p.) injection with 100 µg of ovalbumin (Sigma-ALDRICH, Saint Louis, MO, USA) and 4 mg Inject Alum Adjuvant (Thermo Fisher Scientific, Rockford, IL, USA) in 200 µL of phosphate-buffered saline (PBS) on day 0, 7 and 14. From day 15 to 34, H_2_O and PHF (high dose: 18.4 mg/day, middle dose: 9.2 mg/day, low dose: 4.6 mg/day) in 200 µl H_2_O were intragastrically administrated to the mice. From day 21 to 34, they were challenged by nasal drip with 500 µg OVA in 20 µl PBS an hour after oral PHF treatment. The non-AR control group was sensitized and challenged with PBS and treated with H_2_O. The mice were sacrificed on day 35 (Figure 1).

### 4.3. Evaluation of Nasal Allergic Symptoms

After the last OVA challenge on day 34, the frequencies of sneezing and nose rubbing were counted immediately during the first 10-min period.

### 4.4. Determination of the Proportion of Splenic T Cells

Single cells from the murine spleen were prepared by grinding it, passing it through a 70 µm cell strainer, and suspending it in PBS with 2 mM EDTA and 1% fetal bovine serum (FBS). Red blood cells were lysed by 1× RBC lysis buffer (BioLegend, BD Biosciences, San Jose, CA, USA), and mononuclear cells were washed and cultured in 24-well plates with Roswell Park Memorial Institute (RPMI) 1640 Medium supplemented with 10% fetal bovine serum (FBS) and 1% Penicillin-Streptomycin (5000 U/mL), and then stimulated with 50 ng/mL Phorbol 12-myristate 13-acetate (PMA), 500 ng/mL ionomycin (Sigma-Aldrich) and 1× brefeldin A (Thermo Fisher Scientific) for 5 h followed by FACS staining and analysis. Percentages of CD4^+^ IFN-γ^+^ Th1 cells, CD4^+^ IL-4^+^ Th2 cells, CD4^+^ IL-17^+^ Th17 cells in lymphocytes were analyzed by anti-mouse antibodies against FITC/CD4^+^, PE/IFN-γ^+^, APC/IL-4^+^, PE-Cy7/IL-17^+^ (BioLegend). CD25^high^ FOXP3^+^ Treg cells in lymphocytes were analyzed using Mouse Treg Flow Kit (FOXP3 Alexa Fluor 488/CD4, APC/CD25 PE, BioLegend) by BD FACSVia flow cytometer (BD Biosciences, San Jose, CA, USA).

### 4.5. Collection of Nasal Lavage Fluid (NALF) and Determination of Immune Cells and Nasal Epithelial Cells

NALF was collected after the last intranasal challenge with OVA on day 35. After euthanasia, the trachea was ligated at the upper level and 1 ml cold PBS was gently instilled into the nasopharynx by a 21-gauge catheter. The collections of NALF were centrifuged at 3000 rpm for 5 min at 4 °C. The supernatants were kept at −70 °C until cytokine and chemokine levels were measured. The cells in the NALF were resuspended in 50 µL PBS, centrifuged onto glass slides by the cytospin device (Centrifuge 5403, Eppendorf, Hamburg, Germany), and stained by Kwik Diff Staining kit (Thermo Fisher Scientific). The number of total cells, eosinophils, neutrophils, lymphocytes, macrophages and nasal epithelial cells were counted in five random 200× power fields using a Leica DM6000B microscope (Leica Microsystems GmbH, Wetzlar, Germany) and the Leica Application Suite software (Leica Microsystems GmbH).

### 4.6. Measurement of Immunoglobulin and Cytokine from Serum and NALF

Blood samples were collected by cardiac puncture 24 h after the last OVA challenge on day 35 and serum was collected by centrifugation at 2000× *g* for 20 min at 4 °C. Serum total IgE and OVA-specific IgG1 concentrations were measured using Enzyme-Linked Immunosorbent Assay (ELISA) kit (eBioscience, Hartfield, UK and Cayman Chemical Company, Ann Arbor, MI, USA). Concentrations of murine IL-10, IL-17, TNF-α, CXCL9, and CXCL10 in NALF were measured using Multiplex MAP kit (EMD Millipore Corp., Billerica, MA, USA) using Luminex Bio-plex 200 suspension array system (Bio-Rad Corp. Hercules, CA, USA). 

### 4.7. Histological Examination of Sinonasal Mucosa

The nose of the OVA-induced AR mice was fixed with 4% paraformaldehyde for 24 h and decalcified with 10% ethylenediaminetetraacetic acid (EDTA) for 21 days before being embedded on paraffin. Paraffin sections (5 µm) of sinonasal mucosa tissue were stained with Hematoxylin and Eosin (H&E) (Beyotime Institute of Biotechnology, Haimen, China) for standard histopathological examination. Mucus secretions were stained using the Periodic Acid-Schiff (PAS) staining kit (Sigma-Aldrich) for the determination of goblet cells hyperplasia. The mast cells were stained by Eosinophil-Mast Cell Stain Kit (Abcam, Cambridge, UK). The percentage of goblet cells and the number of infiltrated eosinophils were counted as previously described.

### 4.8. RNA Extraction and Real-Time PCR

Total RNA of mouse nasal mucosa was extracted by RNA extraction kit (Qiagen Corp., Germantown, MD, USA). cDNA was synthesized by Quantiscript Reverse Transcriptase (Qiagen). Quantitative real-time RT-PCR analysis of the cDNA was performed using the LightCycler^®^ 480 Real-Time PCR System (Roche, Basel, Switzerland) with SYBR Green Master Mix (Bio-Rad). The relative mRNA expression level of each gene was determined by using the cycle threshold method (2^−ΔΔCt^) with GAPDH as an internal housekeeping gene. Primer sequences are shown in Table 1.

### 4.9. Data Analysis

All data are presented as mean + standard error of mean (SEM). Results were analyzed by unpaired Student’s *t*-test or nonparametric Mann–Whiney test (equal variances not assumed) using Graph Pad Prism version 5 software (San Diego, CA, USA). Differences were considered statistically significant when *p* value < 0.05.

## 5. Conclusions

Oral treatment of PHF on the OVA-induced AR murine model resulted in immuno-modulatory activities with the suppression of the nasal inflammatory response by reducing the infiltration of eosinophils and mast cells, goblet cell hyperplasia in the nasal epithelium, and the production of the pro-inflammatory cytokine TNF-α and Th2-related cytokines in NALF or the mRNA expression level in the nasal mucosa. PHF can activate Th1 and Treg cells and inhibit Th2 and Th17 cells in the spleen. These results suggested that the PHF could be a potential alternative strategy for the treatment of AR. 

## Figures and Tables

**Table 1:** Primers sequence used in real-time PCR analysis.

Gene	Primer	Oligonucleotide Sequence (5′–3′)
IL-1β	F	5′-CTTCATCTTTGAAGAAGAGCC-3′
	R	5′-CTCTGCAGACTCAAACTCCAC-3′
IL-4	F	5′-ACAGGAGAAGGGACGCCAT-3′
	R	5′-ACCTTGGAAGCCCTACAGA-3′
IL-5	F	5′-TTTGGCACATCCATCTCCG-3′
	R	5′-CGCTCACCGAGCTCTGTTG-3′
IL-6	F	5′-AACGATGATGCACTTGCAGA-3′
	R	5′-GAGCATTGGAAATTGGGGTA-3
IL-10	F	5′-TGGACAACATACTGCTAACCG-3′
	R	5′-GGATCATTTCCGATAAGGCT-3′
IL-13	F	5′-TGAGGAGCTGAGCAACATCACACA-3′
	R	5′-TGCGGTTACAGAGGCCATGCAATA-3′
IL-17	F	5′-GGTCAACCTCAAAGTCTTTAACTC-3′
	R	5′-GGTCAACCTCAAAGTCTTTAACTC-3′
TNF-α	F	5′-CACA GAAAGCATGATCCGCGACGT-3′
	R	5′-CGGCAGAGAGGAGGTTGACTTTCT-3′
TGF-β	F	5′-CACAGAAAGCATGATCCGCGACGT-3′
	R	5′-CGGCAGAGAGGAGGTTGACTTTCT-3
mGAPDH	F	5′-TGGTGAAGCAGGCATCTGAG-3′
	R	5′-TGTTGAAGTCGCAGGAGACAAC-3′

## Data Availability

Not applicable.

## Sample Availability

Samples of the Pentaherbs formula, gallic acid, chlorogenic acid and berberine are available from the authors.
